# Supplementary material for: Molecular Docking, DFT Calculations, Effect of High Energetic Ionizing Radiation, and Biological Evaluation of Some Novel Metal (II) Heteroleptic Complexes Bearing the Thiosemicarbazone Ligand
Source: Molecules. 2021 Sep 27;26(19):5851. doi: 10.3390/molecules26195851 (PMC8512603; doi:10.3390/molecules26195851)
Supplement: Supplementary file 1 [file molecules-26-05851-s001.zip › molecules-1353963-supplementary.pdf]

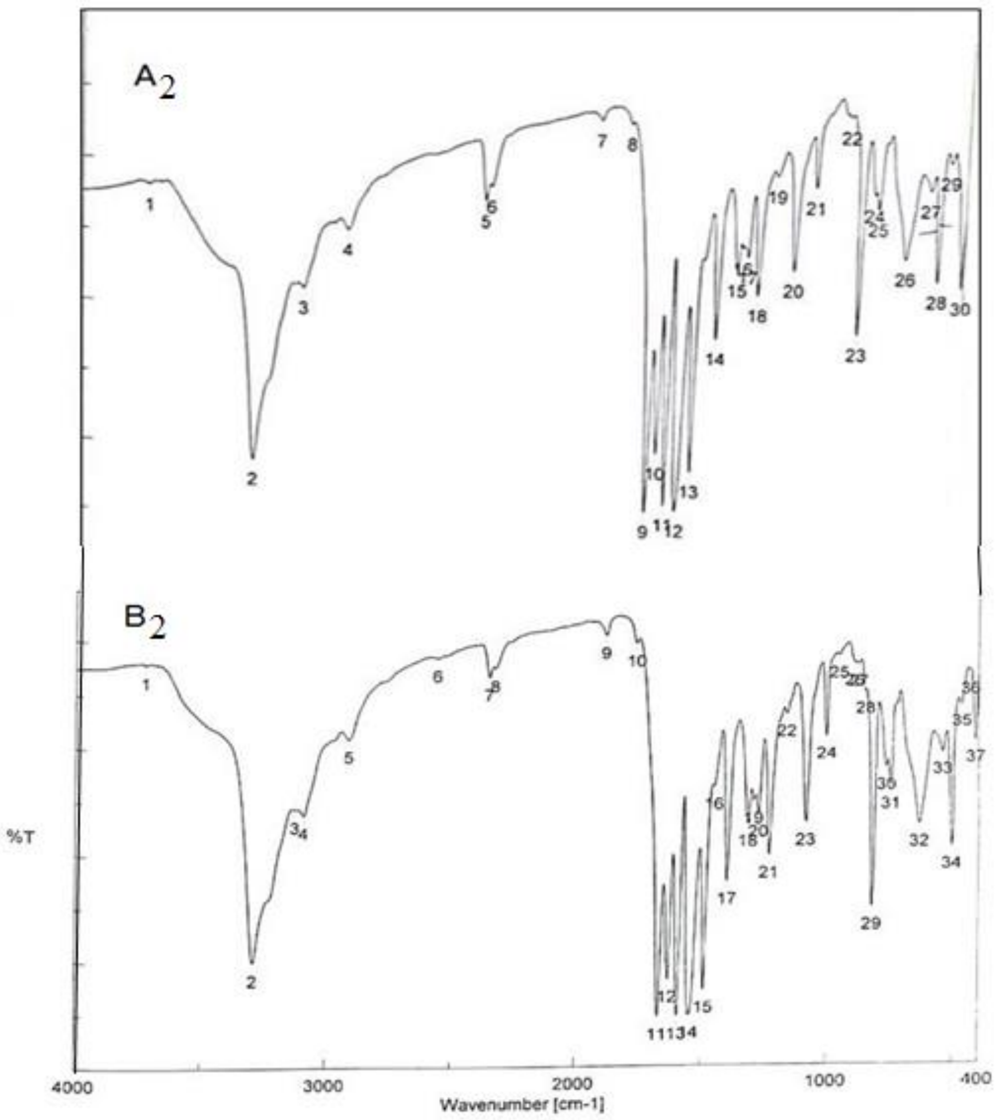

Figure S1a FT-IR spectra of Mn(II) complexes before (B_2) and after (A_2) irradiation.

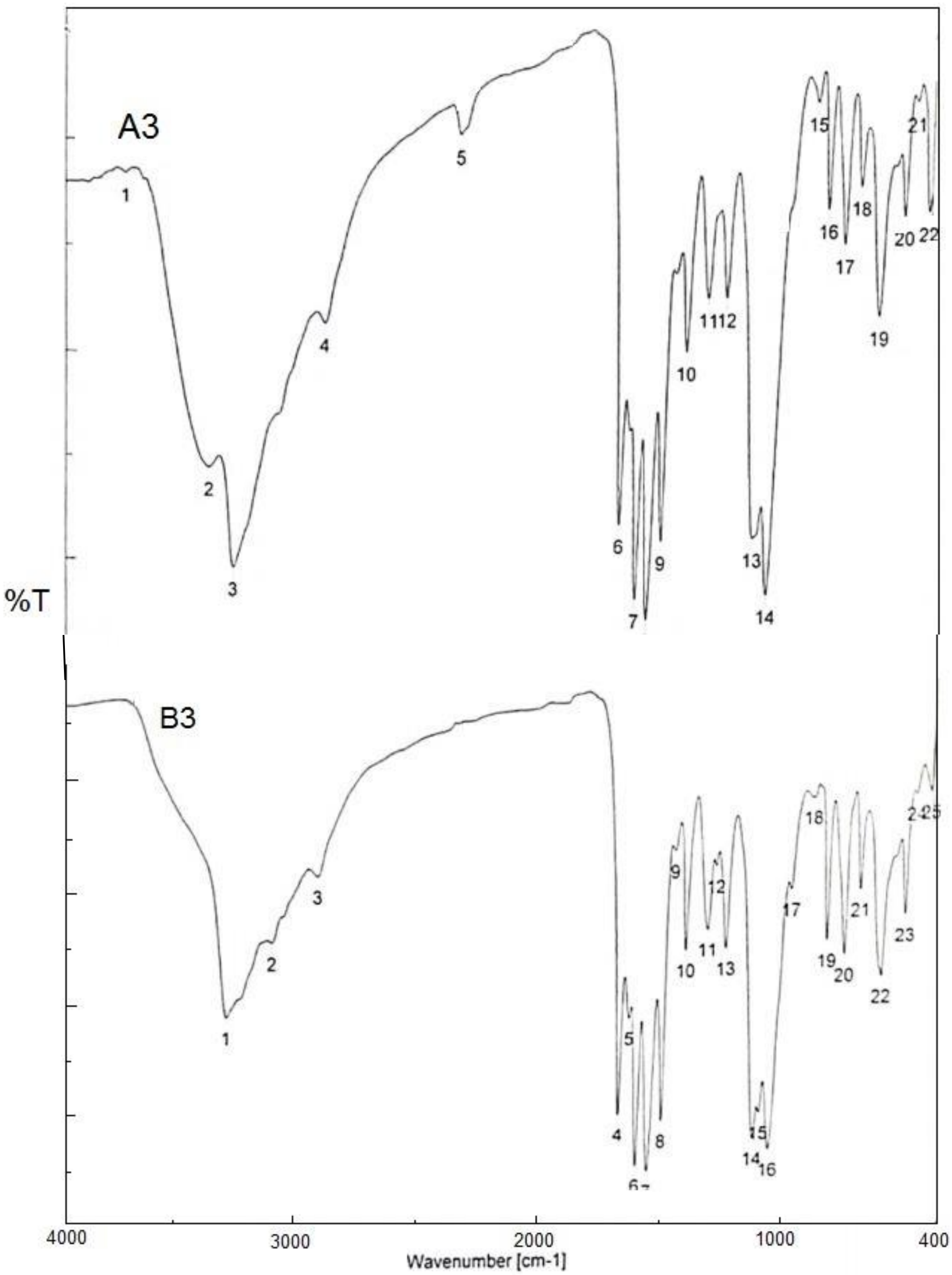

Figure S1b FT-IR of spectra of Hg(II) complexes before (B_3) and after (A_3) irradiation.

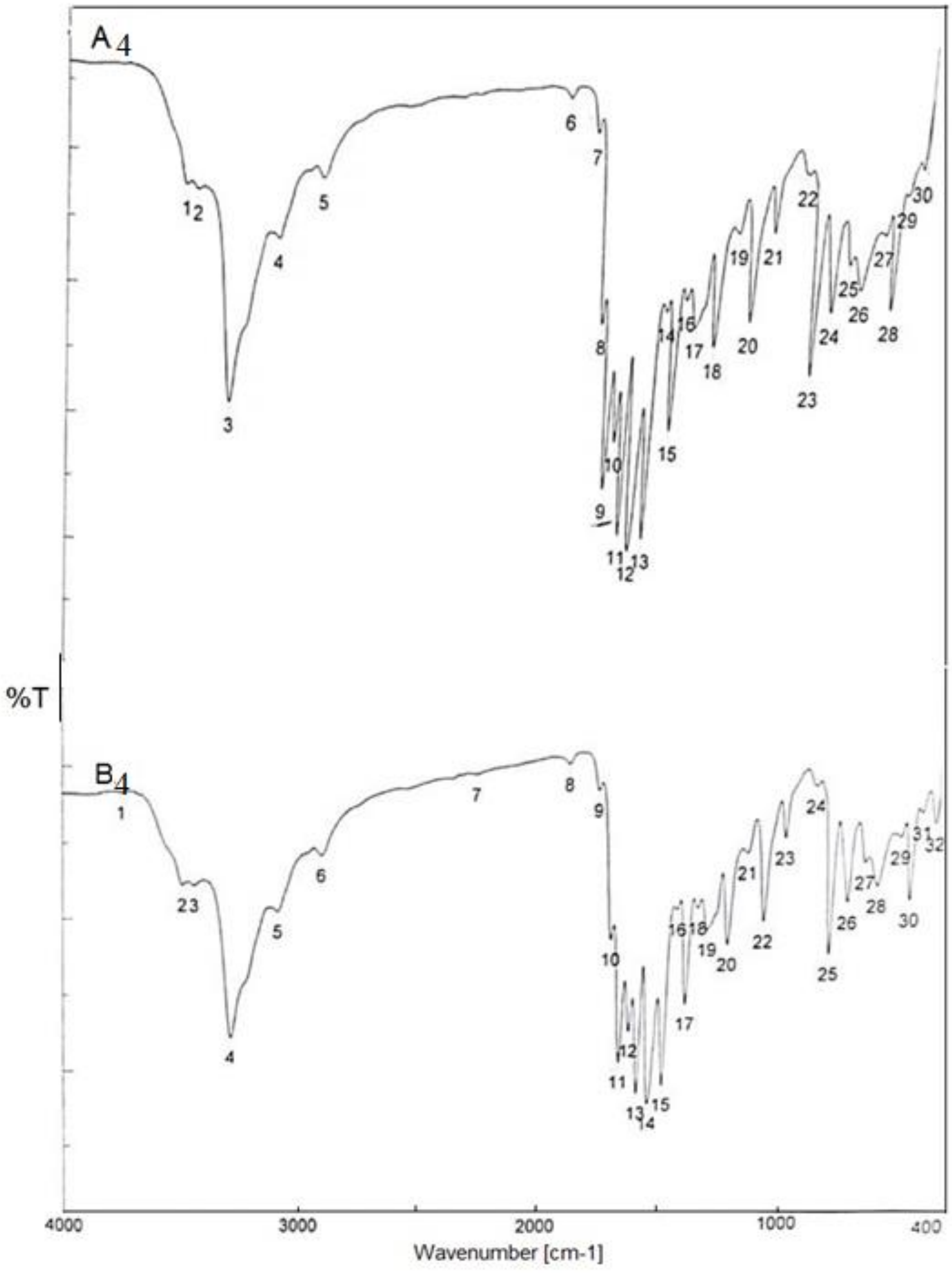

Figure S1c FT-IR of spectra of Zn(II) complexes before (B_4) and after (A_4) irradiation.

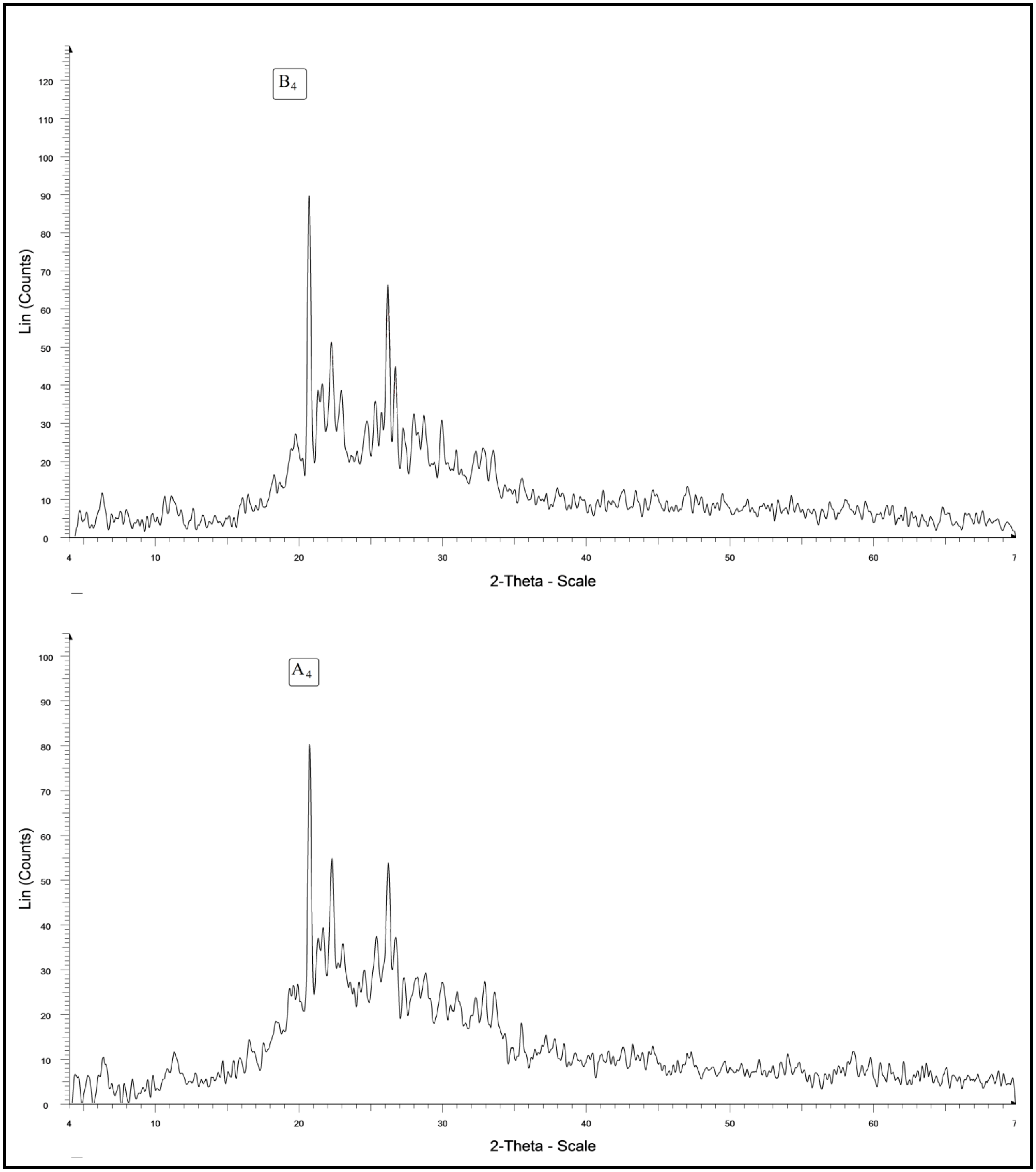

Figure S2: PXRD powder pattern of of Zn(II) complex before (B_4) and after irradiation (A_4).

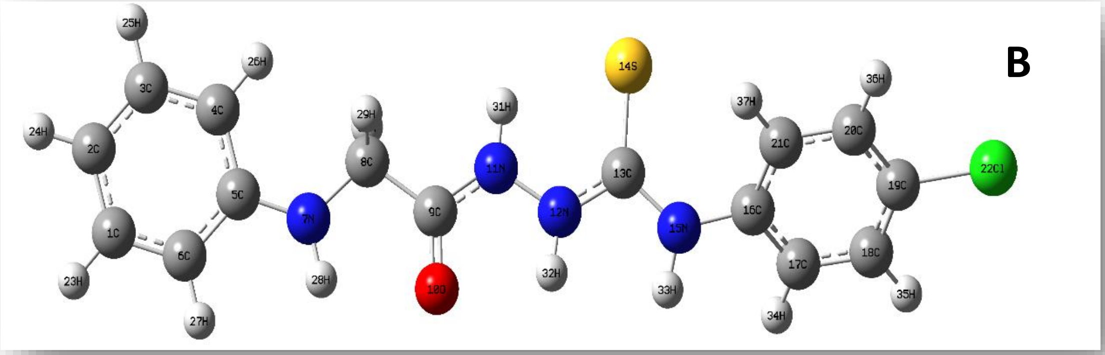
B

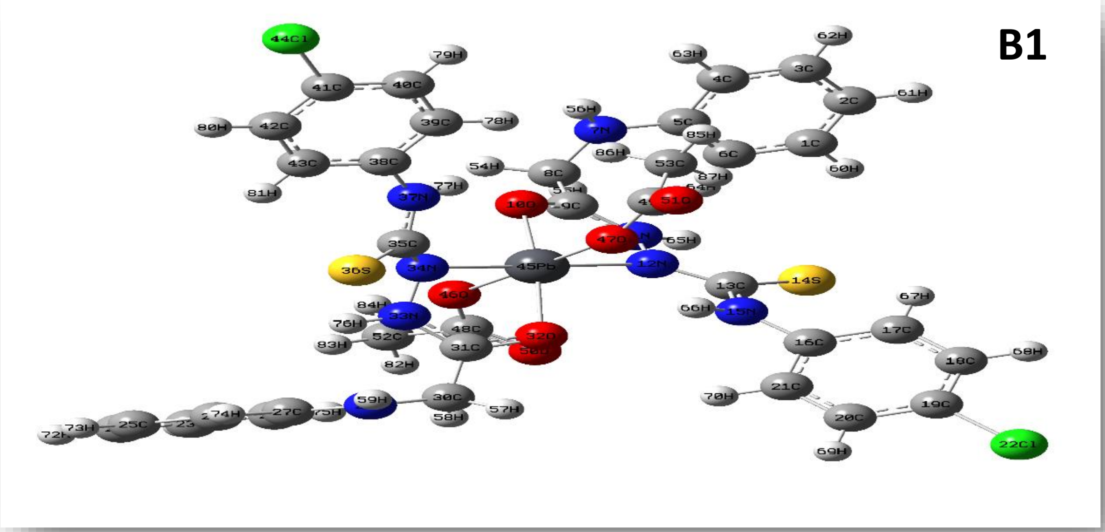
B1

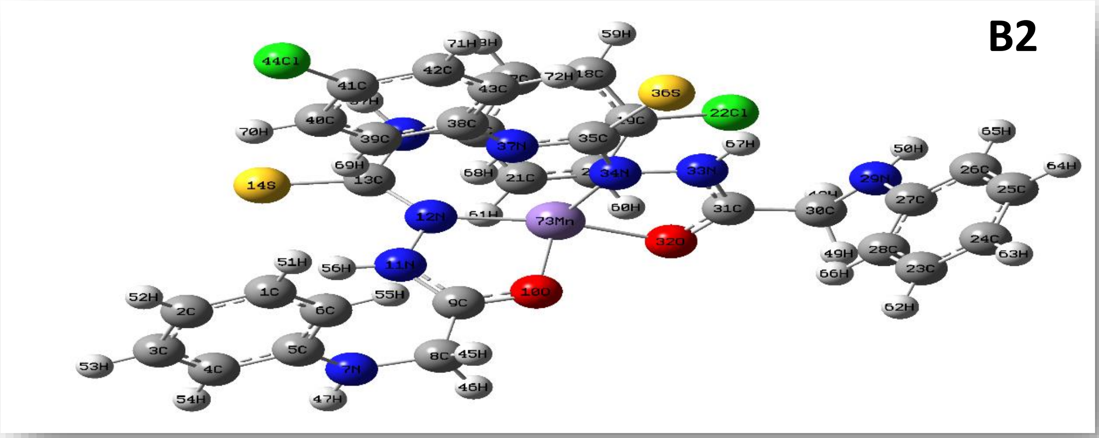
B2

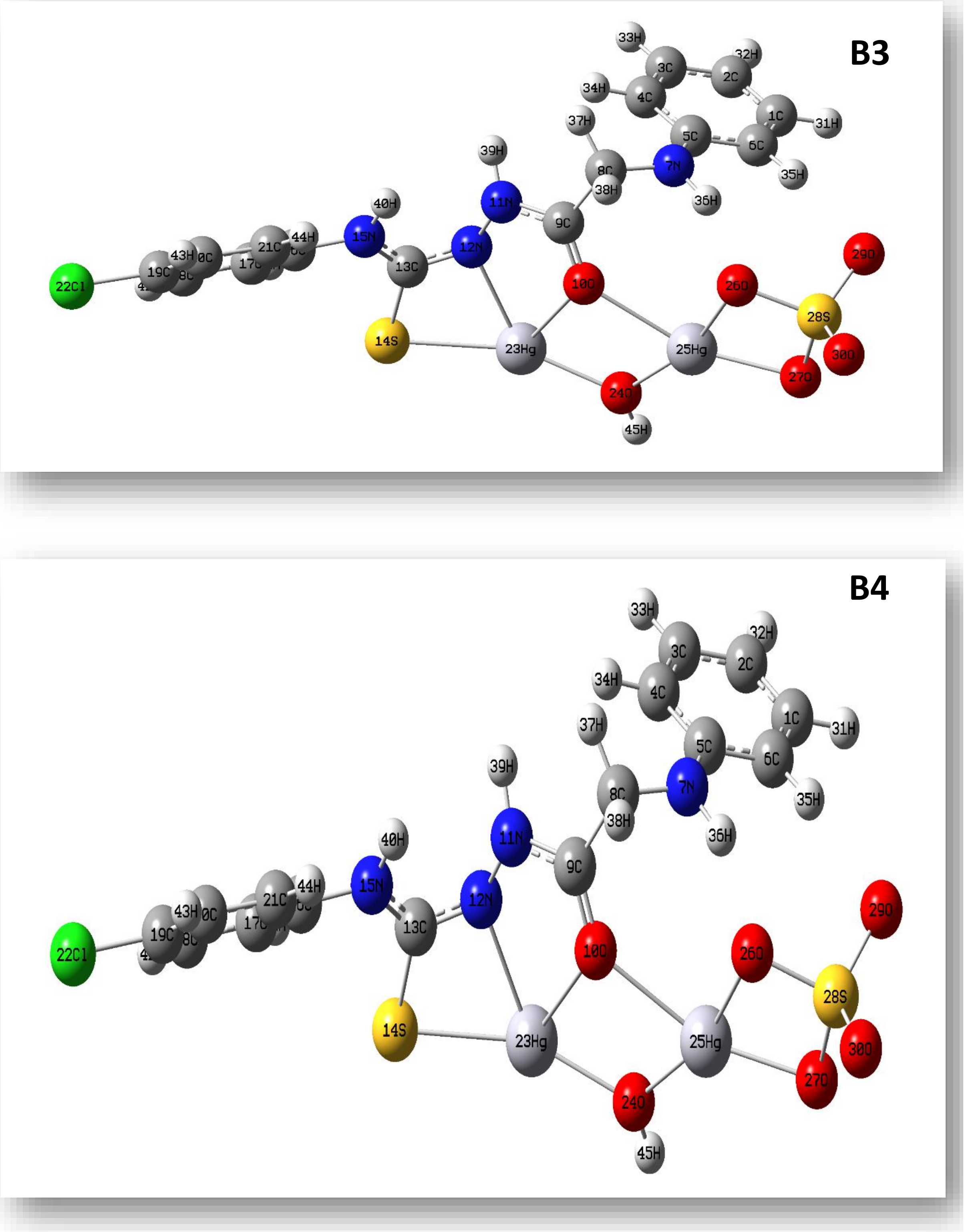

Figure S3. Optimized 3D structures of ligand (B) and the Pb(II) $\mathbf{B_1}$, Mn(II) $\mathbf{B_2}$, Hg(II) $\mathbf{B_3}$, and Zn(II) $\mathbf{B_4}$ complexes.

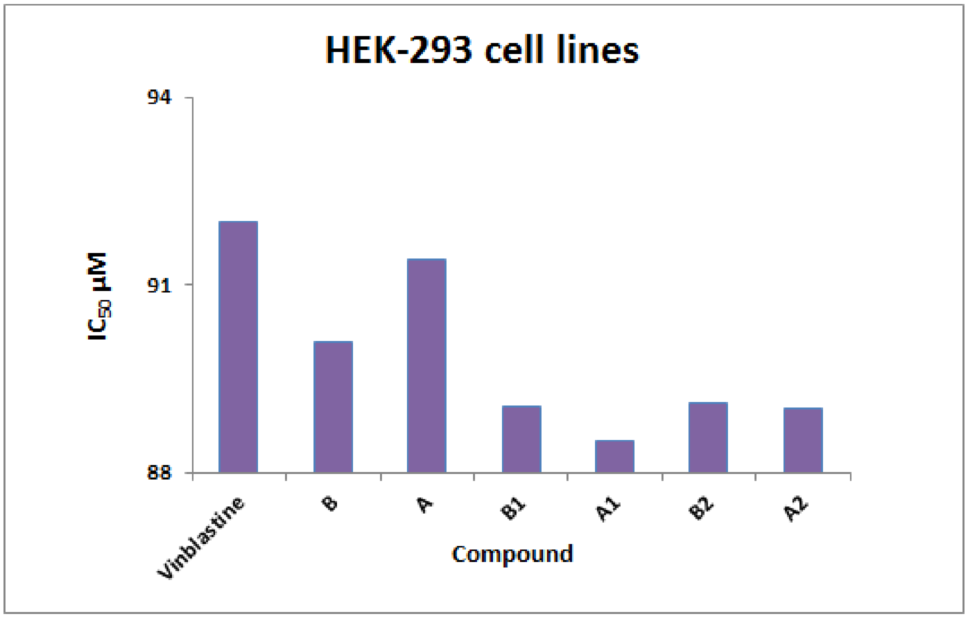

Figure S4: IC_{50} Values of Ligand (B, A) and Mn(II) (B_2, A_2) and Zn(II) (B_4, A_4) complexes before and after irradiation against HEK-293 cell line compared to Vinblastine.

Tables (S1–S5): The coordinates of the optimized ligand (B) and their metal complexes

Table (S1): The coordinates of the optimized ligand (B)

B					
Center	Atomic	Atomic	Coordinates	(Angstroms)	
Number	Number	Type	X	Y	Z
1	6	0	-8.26429	-0.83682	0.157038
2	6	0	-8.64234	0.500823	-0.01845
3	6	0	-7.64769	1.468414	-0.16346
4	6	0	-6.29462	1.120433	-0.13476
5	6	0	-5.90993	-0.22458	0.042706
6	6	0	-6.92245	-1.19956	0.187706
7	7	0	-4.57907	-0.6125	0.07737
8	6	0	-3.46665	0.297044	-0.02326
9	6	0	-2.17919	-0.49941	0.088887
10	8	0	-2.14869	-1.7294	0.351441
11	7	0	-1.02726	0.190131	-0.10481
12	7	0	0.173956	-0.50438	0.02481
13	6	0	1.376399	0.115306	-0.11298
14	16	0	1.477345	1.818999	-0.40226

15	7	0	2.43415	-0.75655	-0.03048
16	6	0	3.829699	-0.46867	-0.00183
17	6	0	4.689612	-1.33252	-0.69395
18	6	0	6.071041	-1.1427	-0.65129
19	6	0	6.575598	-0.07355	0.078122
20	6	0	5.74234	0.798086	0.769387
21	6	0	4.362443	0.594319	0.73749
22	17	0	8.383435	0.192	0.130199
23	1	0	-9.02142	-1.60205	0.270759
24	1	0	-9.68712	0.777667	-0.04134
25	1	0	-7.91982	2.507191	-0.30064
26	1	0	-5.54709	1.892797	-0.25092
27	1	0	-6.64174	-2.23695	0.323449
28	1	0	-4.33742	-1.57573	0.251013
29	1	0	-3.48117	1.0559	0.773384
30	1	0	-3.46943	0.844958	-0.97547
31	1	0	-0.91382	1.177263	-0.30545
32	1	0	0.024035	-1.47499	0.272789
33	1	0	2.213778	-1.74131	-0.08984
34	1	0	4.281834	-2.14663	-1.27899
35	1	0	6.733507	-1.80705	-1.18455
36	1	0	6.156877	1.619348	1.33359
37	1	0	3.710229	1.26026	1.277135

Table (S2): The coordinates of the optimized Pb(II) complex

B1					
Center	Atomic	Atomic	Coordinates	(Angstroms)	
Number	Number	Type	X	Y	Z
1	6	0	5.935198	4.338913	-2.38281
2	6	0	6.170796	5.565814	-1.74071
3	6	0	5.068394	6.361911	-1.36731
4	6	0	3.759795	5.935409	-1.63021
5	6	0	3.517398	4.691908	-2.26741
6	6	0	4.624599	3.89691	-2.64441
7	7	0	2.175998	4.333505	-2.53521
8	6	0	1.739301	3.054904	-3.07261
9	6	0	1.472803	1.989604	-2.01381
10	8	0	0.261004	1.860701	-1.52801
11	7	0	2.438405	1.174106	-1.59381
12	7	0	2.167507	0.246605	-0.57641

13	6	0	3.257708	-0.32819	0.057989
14	16	0	4.896708	0.041811	-0.39871
15	7	0	2.88951	-1.19969	1.044289
16	6	0	3.719212	-2.08189	1.817289
17	6	0	4.859111	-1.63459	2.515489
18	6	0	5.590313	-2.53339	3.311789
19	6	0	5.160416	-3.86309	3.409489
20	6	0	4.024117	-4.32599	2.733189
21	6	0	3.306215	-3.42489	1.927389
22	17	0	6.106119	-5.02529	4.445689
23	6	0	-4.07288	-4.89941	-3.63391
24	6	0	-5.43898	-5.19491	-3.48531
25	6	0	-5.98978	-5.23721	-2.18831
26	6	0	-5.18798	-4.99831	-1.06331
27	6	0	-3.80958	-4.70151	-1.20971
28	6	0	-3.25868	-4.64871	-2.51401
29	7	0	-3.02808	-4.51661	-0.04681
30	6	0	-1.63598	-4.1322	-0.03121
31	6	0	-1.37779	-2.6317	-0.07011
32	8	0	-0.17699	-2.185	0.174589
33	7	0	-2.36369	-1.78961	-0.38521
34	7	0	-2.21409	-0.3687	-0.23411
35	6	0	-3.15309	0.169294	0.673489
36	16	0	-4.39169	-0.79911	1.393089
37	7	0	-3.1086	1.542594	0.769789
38	6	0	-4.0574	2.439492	1.356789
39	6	0	-3.5695	3.603293	1.984089
40	6	0	-4.4551	4.572791	2.486089
41	6	0	-5.8318	4.360288	2.356889
42	6	0	-6.3419	3.211187	1.735889
43	6	0	-5.4519	2.252289	1.226489
44	17	0	-6.9874	5.606185	3.009889
45	82	0	0.001808	-0.0677	-0.45411
46	8	0	-0.29089	-0.7877	-2.36321
47	8	0	0.161406	0.597201	1.448089
48	6	0	0.450711	-1.6609	-3.10861
49	6	0	0.333704	1.663501	2.298189
50	8	0	1.470513	-2.2321	-2.68421
51	8	0	0.022604	1.5778	3.490489
52	6	0	-0.12069	-1.8413	-4.50501
53	6	0	0.959501	2.901602	1.668189
54	1	0	0.793101	3.196602	-3.60391
55	1	0	2.464402	2.688606	-3.80461

56	1	0	1.477597	4.837104	-2.00161
57	1	0	-1.13688	-4.4928	0.877089
58	1	0	-1.08038	-4.5934	-0.85601
59	1	0	-3.52578	-4.46801	0.832589
60	1	0	6.7701	3.707415	-2.67331
61	1	0	7.184095	5.894716	-1.53281
62	1	0	5.229192	7.318312	-0.87711
63	1	0	2.917794	6.569907	-1.35991
64	1	0	4.496601	2.95191	-3.16411
65	1	0	3.440205	1.179308	-1.85901
66	1	0	1.90501	-1.2155	1.298989
67	1	0	5.179109	-0.60429	2.428689
68	1	0	6.469812	-2.19819	3.849989
69	1	0	3.712019	-5.36049	2.821089
70	1	0	2.436816	-3.76339	1.371589
71	1	0	-3.63208	-4.86041	-4.62671
72	1	0	-6.06088	-5.38351	-4.35491
73	1	0	-7.04428	-5.46122	-2.05241
74	1	0	-5.62158	-5.04821	-0.06691
75	1	0	-2.20958	-4.4196	-2.67131
76	1	0	-3.32879	-2.10911	-0.46071
77	1	0	-2.2714	1.994396	0.418889
78	1	0	-2.5024	3.747895	2.114089
79	1	0	-4.076	5.459992	2.980389
80	1	0	-7.4124	3.066484	1.641289
81	1	0	-5.84	1.369188	0.737689
82	1	0	0.486513	-2.5526	-5.06661
83	1	0	-1.15469	-2.1932	-4.43431
84	1	0	-0.14329	-0.8767	-5.02211
85	1	0	1.0242	3.707603	2.400189
86	1	0	0.381201	3.229701	0.796289
87	1	0	1.968502	2.646205	1.322989

Table (S3): The coordinates of the optimized Mn(II) complex

B2					
Center	Atomic	Atomic	Coordinates	(Angstroms)	
Number	Number	Type	X	Y	Z
1	6	0	-5.5558	0.962194	1.956398

2	6	0	-6.8401	0.426994	2.155998
3	6	0	-6.9718	-0.78591	2.862198
4	6	0	-5.8413	-1.45251	3.356098
5	6	0	-4.5466	-0.91201	3.158798
6	6	0	-4.4127	0.303394	2.446698
7	7	0	-3.4259	-1.62431	3.663498
8	6	0	-2.0641	-1.15611	3.620598
9	6	0	-1.3177	-1.41191	2.319498
10	8	0	-0.0105	-1.25591	2.259698
11	7	0	-1.9292	-1.73971	1.190098
12	7	0	-1.0872	-1.82461	0.063398
13	6	0	-1.6736	-2.34681	-1.0754
14	16	0	-3.3377	-2.74451	-1.2629
15	7	0	-0.7733	-2.47141	-2.1391
16	6	0	0.613198	-2.64091	-2.0669
17	6	0	1.394998	-2.37041	-3.219
18	6	0	2.782098	-2.53581	-3.1841
19	6	0	3.416198	-2.98051	-2.0002
20	6	0	2.674898	-3.30121	-0.8708
21	6	0	1.262198	-3.09651	-0.8697
22	17	0	5.218098	-3.14731	-1.9751
23	6	0	4.465299	3.435092	3.350198
24	6	0	5.203999	4.560992	2.949098
25	6	0	5.986699	4.486792	1.778198
26	6	0	6.028899	3.306392	1.021398
27	6	0	5.290799	2.170392	1.429798
28	6	0	4.501399	2.242992	2.601198
29	7	0	5.361599	0.987692	0.638998
30	6	0	4.839799	-0.28551	1.099598
31	6	0	3.359099	-0.45161	0.801498
32	8	0	2.627698	-1.37601	1.329198
33	7	0	2.757999	0.368192	-0.0728
34	7	0	1.371299	0.148393	-0.2541
35	6	0	0.721999	1.207593	-1.0055
36	16	0	1.634799	2.466093	-1.7493
37	7	0	-0.6226	1.020093	-1.039
38	6	0	-1.6886	1.787993	-1.5985
39	6	0	-2.972	1.189294	-1.5213
40	6	0	-4.1014	1.848894	-2.0185
41	6	0	-3.949	3.120894	-2.5932
42	6	0	-2.6909	3.733293	-2.6742
43	6	0	-1.5544	3.069993	-2.1794
44	17	0	-5.404	3.977994	-3.2392

45	1	0	-1.9963	-0.07441	3.820598
46	1	0	-1.4588	-1.63501	4.400398
47	1	0	-3.6148	-2.45321	4.211698
48	1	0	5.363898	-1.10591	0.591498
49	1	0	4.973499	-0.45061	2.178798
50	1	0	6.127599	0.946092	-0.0223
51	1	0	-5.4348	1.900694	1.419598
52	1	0	-7.7183	0.939894	1.775498
53	1	0	-7.9569	-1.21511	3.026198
54	1	0	-5.9577	-2.39021	3.897198
55	1	0	-3.4385	0.756494	2.284298
56	1	0	-2.9247	-1.95351	1.083598
57	1	0	-1.2106	-2.50921	-3.0563
58	1	0	0.918098	-2.02241	-4.1312
59	1	0	3.376098	-2.31511	-4.0659
60	1	0	3.152498	-3.69811	0.018498
61	1	0	0.678698	-3.65851	-0.136
62	1	0	3.859799	3.476292	4.252398
63	1	0	5.1737	5.476392	3.532498
64	1	0	6.5677	5.347492	1.457098
65	1	0	6.639299	3.260892	0.120698
66	1	0	3.923999	1.388892	2.946498
67	1	0	3.193299	1.189292	-0.5124
68	1	0	-0.9364	0.153693	-0.5959
69	1	0	-3.0964	0.197594	-1.0919
70	1	0	-5.0777	1.378694	-1.9677
71	1	0	-2.5887	4.718793	-3.1171
72	1	0	-0.589	3.550193	-2.2508
73	25	0	0.791298	-1.38461	0.540698

Table (S4): The coordinates of the optimized Hg(II) complex

B3					
Center	Atomic	Atomic	Coordinates	(Angstroms)	
Number	Number	Type	X	Y	Z
1	6	0	4.344889	3.402933	-1.64421
2	6	0	3.438098	4.177412	-2.40155
3	6	0	2.126016	4.342606	-1.92634
4	6	0	1.715556	3.739841	-0.72086
5	6	0	2.627005	2.963631	0.04127
6	6	0	3.95677	2.801109	-0.43922

7	7	0	2.27797	2.359274	1.261036
8	6	0	0.919856	2.249492	1.729149
9	6	0	0.0713	1.259539	0.90093
10	8	0	0.478751	0.090324	0.603924
11	7	0	-1.17194	1.673941	0.465318
12	7	0	-1.77285	0.831758	-0.51234
13	6	0	-3.05853	0.446819	-0.40259
14	16	0	-3.46618	-0.96423	-1.47305
15	7	0	-3.92327	1.006502	0.489301
16	6	0	-5.31865	0.738027	0.68714
17	6	0	-6.19469	0.504333	-0.39164
18	6	0	-7.55894	0.272409	-0.14584
19	6	0	-8.03524	0.30168	1.172145
20	6	0	-7.18075	0.554084	2.255195
21	6	0	-5.81493	0.767505	2.007443
22	17	0	-9.79907	0.013758	1.485195
23	80	0	-0.77842	-1.11798	-1.42762
24	8	0	1.227664	-1.97666	-1.33109
25	80	0	2.646959	-1.16959	0.236371
26	8	0	3.557884	0.019217	1.948457
27	8	0	4.829628	-1.71151	0.477703
28	16	0	5.202586	-0.58063	1.756229
29	8	0	6.09849	0.648942	1.182403
30	8	0	5.695793	-1.34838	3.089398
31	1	0	5.366649	3.267375	-1.99106
32	1	0	3.75193	4.642971	-3.33201
33	1	0	1.415314	4.945037	-2.48934
34	1	0	0.699459	3.912309	-0.37091
35	1	0	4.676794	2.21929	0.135317
36	1	0	2.941122	1.676056	1.65824
37	1	0	0.408184	3.223417	1.762552
38	1	0	0.943282	1.855731	2.752766
39	1	0	-1.35389	2.676888	0.422242
40	1	0	-3.49863	1.630687	1.172739
41	1	0	-5.82927	0.507596	-1.41264
42	1	0	-8.23776	0.085802	-0.97139
43	1	0	-7.56595	0.572291	3.269275
44	1	0	-5.14072	0.938528	2.843639
45	1	0	1.540733	-2.66943	-1.94449

Table (S5): The coordinates of the optimized Zn(II) complex

B4					
Center	Atomic	Atomic	Coordinates	(Angstroms)	
Number	Number	Type	X	Y	Z
1	6	0	7.407319	-1.97264	0.2599
2	6	0	7.875331	-3.21043	-0.2317
3	6	0	7.402335	-3.68504	-1.4766
4	6	0	6.483328	-2.93774	-2.218
5	6	0	6.009217	-1.68565	-1.7255
6	6	0	6.482312	-1.21284	-0.4686
7	7	0	5.11081	-0.96176	-2.506
8	6	0	4.494898	0.299437	-2.1298
9	6	0	3.2909	0.122626	-1.2013
10	8	0	2.351908	-0.72618	-1.4853
11	7	0	3.198093	0.875825	-0.1012
12	7	0	2.062694	0.722914	0.7362
13	6	0	2.034786	1.621114	1.777
14	16	0	3.169774	2.947925	1.9624
15	7	0	1.009687	1.485105	2.6921
16	6	0	0.131698	0.373796	2.8947
17	6	0	-1.24171	0.622484	3.1059
18	6	0	-2.1286	-0.44133	3.343
19	6	0	-1.62398	-1.74882	3.351
20	6	0	-0.26088	-2.02191	3.178
21	6	0	0.62291	-0.9505	2.9542
22	17	0	-2.78037	-3.14463	3.5837
23	6	0	-5.18567	-3.34385	-3.3448
24	6	0	-6.54427	-3.26157	-2.9717
25	6	0	-6.89067	-3.25997	-1.5999
26	6	0	-5.90017	-3.34426	-0.619

27	6	0	-4.52637	-3.43345	-0.9925
28	6	0	-4.17857	-3.42544	-2.3745
29	7	0	-3.56507	-3.51744	0.0146
30	6	0	-2.14556	-3.75983	-0.1859
31	6	0	-1.30598	-2.48922	-0.2452
32	8	0	-0.02118	-2.54661	-0.0822
33	7	0	-1.90839	-1.30412	-0.4307
34	7	0	-1.1222	-0.14692	-0.429
35	6	0	-1.82761	1.032378	-0.5734
36	16	0	-3.56081	1.077862	-0.7895
37	7	0	-0.98362	2.125686	-0.485
38	6	0	-1.21323	3.516684	-0.6089
39	6	0	-0.16704	4.362594	-0.1459
40	6	0	-0.28065	5.753093	-0.2369
41	6	0	-1.44656	6.307382	-0.7967
42	6	0	-2.48615	5.491472	-1.2687
43	6	0	-2.37364	4.095573	-1.1821
44	17	0	-1.60027	8.10308	-0.9162
45	30	0	0.848308	-0.7171	-0.115
46	1	0	5.230192	0.971144	-1.6719
47	1	0	4.131094	0.790434	-3.0403
48	1	0	4.749014	-1.41436	-3.3372
49	1	0	-1.75236	-4.34492	0.6528
50	1	0	-1.96076	-4.35222	-1.0897
51	1	0	-3.88577	-3.50454	0.9787
52	1	0	7.766716	-1.60033	1.2155
53	1	0	8.593436	-3.79103	0.3395

54	1	0	7.757444	-4.63553	-1.865
55	1	0	6.129532	-3.30485	- 3.1795
56	1	0	6.140804	-0.26615	- 0.0618
57	1	0	3.850486	1.619431	0.1988
58	1	0	0.94498	2.255904	3.3508
59	1	0	-1.62091	1.63988	3.0658
60	1	0	-3.1847	-0.25013	3.5009
61	1	0	0.113729	-3.039	3.2247
62	1	0	1.688812	-1.14139	2.8733
63	1	0	-4.91337	-3.33945	- 4.3966
64	1	0	-7.31767	-3.19777	- 3.7311
65	1	0	-7.93397	-3.19588	- 1.3045
66	1	0	-6.17197	-3.34806	0.4346
67	1	0	-3.14107	-3.47733	- 2.6904
68	1	0	-2.92289	-1.15783	- 0.5538
69	1	0	-0.02302	1.897095	- 0.2301
70	1	0	0.732564	3.937702	0.2967
71	1	0	0.517041	6.3937	0.1239
72	1	0	-3.37335	5.935664	- 1.7079
73	1	0	-3.17223	3.468766	- 1.5538

Table S6: Antimicrobial activity of unirradiated and irradiated complexes

No	Compound	Inhibition zone diameter/mm mg^{-1} sample						
		G -			G +		Fungi	
		K. pneumonia	***E. coli***	***P. aeruginosa***	***S. mutans***	***S. aureus***	***C. albicans***	***A.Nigar***
Gentamicin		25±0.5	27±0.5	30±0.5	-	-	-	-
Ampicillin		-	-	-	30±0.5	22±0.1	-	-
Nystatin		-	-	-	-	-	21±0.5	19±0.5
B	H_2L	15.3±0.5	15.3±0.6	15.3±0.5	NA	13.6±0.5	NA	NA
A	H_2L	17.6±0.5	14.3±0.5	NA	NA	11.6±0.5	NA	NA
B_1	$[Pb(H_2L)_2(OAc)_2]ETOH.H_2O$	NA	NA	NA	NA	NA	NA	NA
A_1	$[Pb(H_2L)_2(OAc)_2]ETOH.H_2O$	11.6±0.5	NA	NA	NA	10.3±0.5	NA	10.6±0.5
B_2	$[Mn(H_2L)(HL)]Cl$	NA	NA	NA	NA	NA	NA	NA
A_2	$[Mn(H_2L)(HL)]Cl$	12.6±0.5	NA	NA	NA	10.6±0.5	NA	NA

B_3	$[Hg_2(H_2L)(OH)SO_4]$	22.6±0.6	23.3±0.6	21.3±0.6	28.6±0.6	36.6±0.6	23.6±0.6	29.6±0.6
A_3	$[Hg_2(H_2L)(OH)SO_4]$	20.6±0.6	29.6±0.6	27.6±0.6	19.6±0.6	33.3±0.6	31.6±0.6	30.6±0.6
B_4	$[Zn(H_2L)(HL)]$	NA	NA	NA	NA	NA	10.3±0.5	NA
A_4	$[Zn(H_2L)(HL)]$	NA	NA	NA	NA	13.3±0.5	NA	24.6±0.6

- NA: No activity
